# Digital twins to personalize medicine

**DOI:** 10.1186/s13073-019-0701-3

**Published:** 2019-12-31

**Authors:** Bergthor Björnsson, Carl Borrebaeck, Nils Elander, Thomas Gasslander, Danuta R. Gawel, Mika Gustafsson, Rebecka Jörnsten, Eun Jung Lee, Xinxiu Li, Sandra Lilja, David Martínez-Enguita, Andreas Matussek, Per Sandström, Samuel Schäfer, Margaretha Stenmarker, X. F. Sun, Oleg Sysoev, Huan Zhang, Mikael Benson, on behalf of the Swedish Digital Twin Consortium

**Affiliations:** 1 Department of Surgery and Clinical and Experimental Medicine, Linköping University, 581 83 Linköping, Sweden; 2 Department of Immunotechnology, Lund University, Medicon Village, Scheelevägen, Lund, Sweden; 3 Departments of Oncology, and Clinical and Experimental Medicine, Linköping University, 581 83 Linköping, Sweden; 4 Centre for Personalized Medicine, Linköping University, 581 83 Linköping, Sweden; 5 Bioinformatics, Department of Physics, Chemistry and Biology, Linköping University, 581 83 Linköping, Sweden; 6 Mathematical Sciences, University of Gothenburg and Chalmers University of Technology, 412 96 Gothenburg, Sweden; 7 Department of Otorhinolaryngology, Yonsei University College of Medicine, Seoul, South Korea; 8 Division of Clinical Microbiology, Department of Laboratory Medicine, Karolinska Institutet, Karolinska University Hospital, 141 52 Huddinge, Stockholm, Sweden; 9 Department of Laboratory Medicine, Region Jönköping County, Jönköping, Sweden; 10 Futurum–Academy for Health and Care, Department of Pediatrics, Region Jönköping County, Jönköping, Sweden; 11 Department of Pediatrics, Institution for Clinical Sciences, 413 90 Göteborg, Sweden; 12 Division of Statistics and Machine Learning, Department of Computer and Information Science, Linköping University, 581 83 Linköping, Sweden; 13 Crown Princess Victoria Children’s Hospital, 581 85 Linköping, Sweden; 14 Wallenberg Centre for Molecular Medicine, Linköping University, 581 83 Linköping, Sweden

## Abstract

Personalized medicine requires the integration and processing of vast amounts of data. Here, we propose a solution to this challenge that is based on constructing Digital Twins. These are high-resolution models of individual patients that are computationally treated with thousands of drugs to find the drug that is optimal for the patient.

## Background

Despite great strides in biomedical advances during the past century, a large number of patients do not respond to drug treatment. According to a report from the US Food and Drug Administration (FDA), medication is deemed ineffective for 38–75% of patients with common diseases [1]. This results in patient suffering and increased healthcare costs. These problems reflect the complexity of common diseases, which may involve altered interactions between thousands of genes that differ between patients with the same diagnosis. There is a wide gap between this complexity and modern health care, in which diagnostics often relies on a small number of biomarkers of limited sensitivity or specificity. Digital and genomic medicine may bridge this gap by monitoring, processing, and integrating vast amounts of data from wearable digital devices, omics, imaging, and electronic medical records [2]. However, the integration and clinical exploitation of such complex data are unresolved challenges.

## Application of the digital twin concept to personalize medicine

**Fig. 1:**
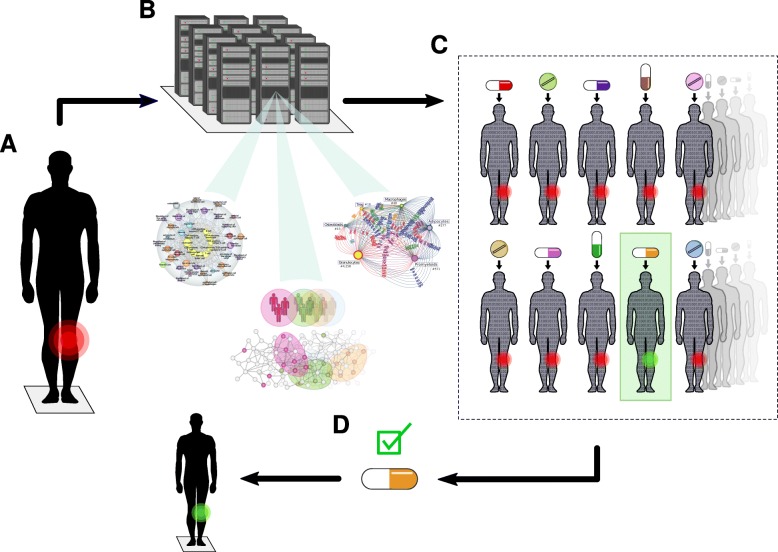
The digital twin concept for personalized medicine. **a** An individual patient has a local sign of disease (*red*). **b** A digital twin of this patient is constructed in unlimited copies, based on computational network models of thousands of disease-relevant variables. **c** Each twin is computationally treated with one or more of the thousands of drugs. This results in digital cure of one patient (*green*). **d** The drug that has the best effect on the digital twin is selected for treatment of the patient

Digital twins are a concept from engineering which has been applied to complex systems such as airplanes or even cities [3]. The aims are to model those systems computationally, in order to develop and test them more quickly and economically than is possible in the real-life setting. Ideally, the digital twin concept can be translated to patients in order to improve diagnostics and treatment. This is the general aim of the DigiTwin consortium, which includes academic, clinical and industrial partners from 32 countries (https://www.digitwins.org). Practical and scalable solutions for specific problems will also require national initiatives. As an example, the Swedish Digital Twin Consortium (SDTC) aims to develop a strategy for personalized medicine (https://www.sdtc.se). The SDTC strategy, which is the focus of this Comment, is based on: (i) constructing unlimited copies of network models of all molecular, phenotypic, and environmental factors relevant to disease mechanisms in individual patients (i.e., digital twins); (ii) computationally treating those digital twins with thousands of drugs in order to identify the best performing drug; and (iii) treating the patient with this drug (Fig. 1).

Clinical implementation of this strategy has presented questions that must be addressed: Which information is needed? How can it be integrated and analyzed? If we start with the molecular changes, these are dispersed across an unknown number of cell types in the body. A recent study indicated that 50% of 45 analyzed cell types were involved in each of more than 100 diseases [4]. Can we analyze all those cell types simultaneously in patients? If we look at an inflammatory disease, rheumatoid arthritis, many of the cell types are located in tissues that are difficult to obtain from patients, such as the liver or lungs. However, it is possible to perform multi-omics analyses of individual cells from even small quantities of any fluid or tissue that can be obtained from the body. For example, single-cell RNA-sequencing (scRNA-seq) has been used to profile the mRNA in thousands of cells in many diseases. This has already resulted in the identification of novel mechanisms that can potentially be exploited for personalized medicine [5, 6]. However, the complexity of those mechanisms makes drug prioritization a formidable challenge. For example, scRNA-seq analysis of inflammatory and malignant diseases implicated hundreds of drugs, many of which targeted mechanisms that did not overlap [4]. Thus, targeting one mechanism may not be effective. How can we integrate and analyze all the data derived from scRNA-seq to prioritize mechanisms for drug treatment?

## Network tools to construct and exploit digital twins for personalized medicine

A large body of evidence suggests that complex systems can be described and analyzed by network tools. In the context of medicine, protein–protein interaction (PPI) networks can be used as templates, to which disease-associated genes can be mapped [7, 8].

Such variables tend to co-localize and form modules which contain the genes that are most important for pathogenesis, diagnostics, and therapeutics [8]. Other network tools can be applied to prioritize individual genes in a module. For example, the most interconnected, or central, nodes tend to be most important. We propose that the same methods can be applied to construct digital twins of individual patients.

## Expanding digital twins by integrating variables of multiple types, locations, and time points

A digital twin should ideally integrate all of the types of variable that are relevant to pathogenesis. If the variables are different types of molecules, these can be mapped on the PPI network in order to form multilayer modules [8]. Consider, for example, one module formed by mRNAs and another formed by genes harboring disease-associated variants. If the mRNAs and genes map to the same proteins, the two modules can be linked. The same principle can be applied to integrate many other types of molecules, such as mRNAs or proteins.

The multilayer modules can be used to form and test hypotheses, which may have direct implications for translating diagnostics and the treatment of a digital twin to patient care. For example, if a disease-associated single nucleotide polymorphism (SNP) causes the altered expression of a protein in a twin, this would lead to in silico treatment with a drug that specifically blocks that protein. If successful, this could, in turn, motivate diagnostic measurement of the protein in the patient. If the protein level is elevated, the patient would be treated with the same drug.

However, diagnostic and therapeutic decisions generally need to consider multiple types of data other than molecules, such as symptoms or environmental factors, which means that the digital twin concept cannot be restricted to molecular profiles. As an example, in severe asthma, a combination of allergen avoidance and medication may be needed. An important advantage of multilayer modules is that they can potentially integrate molecular modules with modules representing other types of disease-relevant data. For example, symptoms from multiple diseases can be linked into a network that is based on co-occurrence, and form modules (that represent wheezing and coughing in asthma). Such phenotypic modules can be linked to their corresponding molecular modules [7, 8]. With increasing availability of multi-omics, phenotypic, and environmental data, network tools may allow the construction of disease models of unprecedented resolution. Such models may serve as templates for the construction of digital twins for individual patients.

Network tools can also be used to link interactions between cell types in different tissues. For example, cells in an arthritic joint may interact with cells in adjacent lymph nodes through different mediators [4]. Thus, multicellular network models from different tissues may be linked into a meta-network of interacting models, thereby generating comprehensive digital twins. Network tools, such as centrality, can then be applied to prioritize the most important tissues, cell types, and genes. This is important because causal mechanisms may reside in tissues other than those that cause symptoms. For example, in rheumatoid arthritis, the lungs have been proposed to have such a role and might be more suitable for therapeutic targeting than joints. The same principles can be applied to link tissues and cells over time [9]. This is important because many diseases evolve over many years before symptoms and diagnosis occur, by which time treatment may be unsuccessful because of irreversible tissue damage. Therefore, early diagnosis and treatment are important. Taken together, network tools may be exploited to construct high-resolution twins that enable the prioritization of biomarkers and drug targets for personalized medicine, even if the causal cell types are not accessible for analysis. It is also important to recognize that other methods, such as machine learning and artificial intelligence, can be used complementarily to construct and analyze digital twins. Examples include modeling the development of the networks over time or predicting the optimal treatments from the network structures. In this scenario, the digital twin model can be considered as an artificial intelligence system that interacts with the drugs and experiences the changes that occur in the human body. Various machine-learning tools, such as Bayesian Networks, Deep Learning, Decision Trees, Causal Inference, or State-Space models, may be needed [10].

## Conclusions

The clinical implementation of digital twins will require solving a wide range of technical, medical, ethical, and theoretical challenges. The costs and complexity will be comparable to those of projects such as the Human Genome Project (HGP), but may lead not only to greatly improved health care and understanding of disease mechanisms but also to completely new research directions. Another potential similarity to HGP could be the potential to inspire technical developments, leading to a decrease in both the costs and the difficulties involved in clinically implementing digital twins. Given the importance of the medical problem, the potential of digital twins merits concerted research efforts on a scale similar to those involved in the HGP.

## Abbreviations

HGP: Human Genome Project
PPI: protein–protein interaction
scRNA-seq: Single-cell RNA-sequencing
SDTC: Swedish Digital Twin Consortium
